# Clinical effectiveness of septoplasty versus medical management for nasal airways obstruction: multicentre, open label, randomised controlled trial

**DOI:** 10.1136/bmj-2023-075445

**Published:** 2023-10-18

**Authors:** Sean Carrie, James O’Hara, Tony Fouweather, Tara Homer, Nikki Rousseau, Leila Rooshenas, Alison Bray, Deborah D Stocken, Laura Ternent, Katherine Rennie, Emma Clark, Nichola Waugh, Alison J Steel, Jemima Dooley, Michael Drinnan, David Hamilton, Kelly Lloyd, Yemi Oluboyede, Caroline Wilson, Quentin Gardiner, Naveed Kara, Sadie Khwaja, Samuel C Leong, Sangeeta Maini, Jillian Morrison, Paul Nix, Janet A Wilson, M Dawn Teare

**Affiliations:** 1 Department of Ear, Nose and Throat, Newcastle Upon Tyne Hospitals NHS Foundation Trust, Freeman Hospital, High Heaton, Newcastle upon Tyne, NE7 7DN, UK; 2 Population Health Sciences Institute, Newcastle University, Newcastle upon Tyne, UK; 3 Leeds Institute of Clinical Trials Research, University of Leeds, Leeds, UK; 4 Bristol Medical School, Population Health Science Institute, University of Bristol, Bristol, UK; 5 Northern Medical Physics and Clinical Engineering, Newcastle Upon Tyne Hospitals NHS Foundation Trust, Newcastle upon Tyne, UK; 6 Translational and Clinical Research Institute, Newcastle University, Newcastle upon Tyne, UK; 7 Newcastle Clinical Trials Unit, Newcastle University, Newcastle upon Tyne, UK; 8 Centre for Academic Primary Care, University of Bristol, Bristol, UK; 9 Northern Ireland Clinical Trials, Belfast, Northern Ireland, UK; 10 Department of Ear, Nose and Throat, NHS Tayside, Dundee, UK; 11 Department of Ear, Nose and Throat, County Durham and Darlington NHS Foundation Trust, Durham, UK; 12 Department of Ear, Nose and Throat, Manchester University Foundation NHS Trust, Manchester, UK; 13 Department of Ear, Nose and Throat, Liverpool University Hospitals NHS Foundation Trust, Liverpool, UK; 14 Department of Ear, Nose and Throat, NHS Grampian, Aberdeen, UK; 15 General Practice and primary Care, University of Glasgow, Glasgow, UK; 16 Department of Ear, Nose and Throat, Leeds Teaching Hospitals NHS Trust, Leeds, UK

## Abstract

**Objective:**

To assess the clinical effectiveness of septoplasty.

**Design:**

Multicentre, randomised controlled trial.

**Setting:**

17 otolaryngology clinics in the UK’s National Health Service.

**Participants:**

378 adults (≥18 years, 67% men) newly referred with symptoms of nasal obstruction associated with septal deviation and at least moderate symptoms of nasal obstruction (score >30 on the Nasal Obstruction and Symptom Evaluation (NOSE) scale).

**Interventions:**

Participants were randomised 1:1 to receive either septoplasty (n=188) or defined medical management (n=190, nasal steroid and saline spray for six months), stratified by baseline symptom severity and sex.

**Main outcome measures:**

The primary outcome measure was patient reported score on the Sino-Nasal Outcome Test-22 (SNOT-22) at six months, with 9 points defined as the minimal clinically important difference. Secondary outcomes included quality of life and objective nasal airflow measures.

**Results:**

Mean SNOT-22 scores at six months were 19.9 (95% confidence interval 17.0 to 22.7) in the septoplasty arm (n=152, intention-to-treat population) and 39.5 (36.1 to 42.9) in the medical management arm (n=155); an estimated 20.0 points lower (better) for participants randomised to receive septoplasty (95% confidence interval 16.4 to 23.6, P<0.001, adjusted for baseline continuous SNOT-22 score and the stratification variables sex and baseline NOSE severity categories). Greater improvement in SNOT-22 scores was predicted by higher baseline symptom severity scores. Quality of life outcomes and nasal airflow measures (including peak nasal inspiratory flow and absolute inhalational nasal partitioning ratio) improved more in participants in the septoplasty group. Readmission to hospital with bleeding after septoplasty occurred in seven participants (4% of 174 who had septoplasty), and a further 20 participants (12%) required antibiotics for infections.

**Conclusions:**

Septoplasty is a more effective intervention than a defined medical management regimen with a nasal steroid and saline spray in adults with nasal obstruction associated with a deviated nasal septum.

**Trial registration:**

ISRCTN Registry ISRCTN16168569.

## Introduction

Septoplasty is a common operation to alleviate nasal obstruction associated with deviation of the nasal septum. It may be accompanied by reduction of the inferior turbinate to increase airflow through the nasal cavities. Surgery is typically predicated on clinical history and a visual assessment of the nasal septum. To date, no objective nasal airflow measures have proven definitively beneficial in the selection of patients. Despite the lack of an evidence base, in 2019/20 about 16 700 septoplasty procedures were performed in England^1^ at an estimated cost of £15.9m ($19.9m; €18.5m) for the operations alone. More than 250 000 septoplasties are performed in the United States annually.^2^

Many UK clinical commissioning guidelines propose a trial of medical treatment before surgical referral.^3^ Medical treatment usually entails intranasal steroid spray, although recommendations on dose and length of treatment are inconsistent. Some National Health Service commissioning bodies in England have placed septoplasty on a list of restricted treatments. Other healthcare providers will only routinely fund septoplasty for nasal obstruction that is causing documented medical problems, such as sleep or breathing disruption, and only after all non-surgical treatments have been tried.^4^

The Nasal Airways Obstruction Study (NAIROS) was designed to provide definitive evidence and recommendations for the clinical effectiveness of septoplasty, to inform guidance on which patients may benefit from this treatment, and to standardise treatment across the UK.

## Methods

The Nasal Airways Obstruction Study was a multicentre, non-adaptive, open label randomised controlled trial conducted in 17 hospitals in the UK’s NHS. The regional ethics committee approved the trial protocol.^5^ Independent data monitoring and trials steering committees oversaw the trial.

### Participants

Participants were adults (≥18 years) newly referred to otolaryngology clinics with symptoms of nasal obstruction associated with septal deviation and confirmed by endoscopic assessment. Participants were offered entry into the trial if their presenting Nasal Obstruction and Symptom Evaluation (NOSE)^6^ score was ≥30 (out of a potential score of 100), with 30 defined as the cut-off to differentiate patients with nasal obstruction from those without.^7^ We excluded participants with relevant systemic inflammatory diseases or previous septal surgery (see supplementary material for inclusion and exclusion criteria). All participants provided written informed consent.

### Randomisation

The Newcastle Clinical Trials Unit Randomisation Service, an in-house bespoke internet based system, performed randomisation centrally using permuted random blocks of variable length. All allocations were generated by an automated process within the system. Participants were randomised 1:1 to receive either surgical intervention or medical management, stratified by sex and baseline severity of symptoms according to scores on the NOSE scale: 30-50 (moderate), 55-75 (severe), 80-100 (extreme).^7^ Open label randomisation was administered centrally through the Newcastle University Clinical Trials Unit, using a secure web based system.

### Interventions

*Septoplasty*—Participants randomised to septoplasty received surgery up to 12 weeks post-randomisation. The surgery was encouraged to take place within eight weeks of randomisation, with a further four weeks permissible for extenuating circumstances. Experienced surgeons not in training performed the surgery. Because septoplasty is a commonly performed procedure, data on individual surgeons was not collected, with the surgical team represented by the recruitment site within the analysis. As a pragmatic study, we permitted surgery for unilateral inferior turbinate reduction on the wider side at the discretion of the surgeon, reflecting the considerable variation in current UK surgical practice. Deviation of the septum often causes bilateral nasal obstruction as a result of the deviation blocking one side and the inferior turbinate becoming enlarged in the opposite nasal cavity. Surgical methods employed were the same as those used in standard surgical care at recruitment sites—the only stipulations being that the surgery had to be through a closed approach (that is, no external incisions), and no grafts were to be used. Surgeons were, however, allowed to adopt a variety of techniques to manipulate the nasal septum cartilage into place, and so overall results reflect the generality of current surgical practice. The only stipulation for the method used to perform the inferior turbinate reduction was that mucosal preserving techniques should be undertaken.

*Medical management*—Participants randomised to receive medical management were supplied with six months’ supply of isotonic nasal saline spray (Sterimar; Sofibel, Paris, France) to be used twice daily, and mometasone furoate nasal steroid spray to be used twice daily after the saline spray and then 100 μg twice daily for six weeks, then reduced to 100 μg once daily or 50 μg twice daily for the remainder of the six months. Participants randomised to receive medical management were assured they would have the opportunity to discuss the option of septoplasty after the six month primary outcome time point.

### Outcome measures

#### Primary outcome measure

The primary outcome measure was the score on the Sino-Nasal Outcome Test (SNOT-22) at six months (defined protocol time window −2 to 4 weeks for completion). SNOT-22 is a patient reported outcome measure that has been validated to assess symptoms related to chronic rhinosinusitis^8^ but has been used to assess outcomes after septal surgery.^9^ SNOT-22 comprises 22 items, each scored from zero to 5. The total score ranges from zero to 110, with higher scores indicating worse symptoms.

#### Secondary outcome measures

Four secondary patient reported outcome measures were assessed:

• SNOT-22 score at 12 months.

• SNOT-22 subscale (nose, sleep, ear/facial pain, psychological) score at baseline, six months, and 12 months.^8^

• Score on the NOSE scale at baseline, six months, and 12 months.^6^ This scale comprises five items, each scored from zero to 4. The total score is multiplied by 5 to give an overall range from zero to 100, with higher scores indicating worse symptoms.

• General quality of life, measured at baseline, six months, and 12 months using the physical component scores and mental component scores of the 36 item Short Form survey (SF-36), which assesses recall over the preceding week, with higher scores indicating better quality of life.^10^ The scores are transformed into a range from zero to 100, with zero representing the worst possible health and 100 representing the best possible health.

Secondary clinical measures at baseline, six months, and 12 months were:

• The double ordinal airway subjective scale, which is a subjective comparator of right and left nasal patency,^11^ with each nostril rated from 1 to 10, with higher scores indicating better airflow. The results are presented as the modulus of the absolute subjective double ordinal airway subjective scale=(left score−right score)/left score+right score)—that is, ignoring positive or negative results. Scores range from 0 to 1; scores closer to 0 indicating symmetrical nasal airways. 

• Objective assessments of nasal patency after decongestant use, including peak nasal inspiratory flow, measured using a peak nasal inspiratory flow meter (GM Instruments, Kilwinning, UK).^12^ Nasal partitioning ratio (using mean volumes from three maximal inhalation measurements, scored 0 to ±1, with 0 indicating symmetrical nasal airways and ±1 indicating complete left side (+1) and right side (−1) unilateral airflow), tidal volume, and maximal inhalation flow rate measured using a rhinospirometer (NV1 rhinospirometer; GM Instruments, Kilwinning, UK).

• An imbedded economic evaluation is to be reported separately in a future publication.

### Statistical analysis

Analyses were performed according to a predetermined analysis plan.^13^ Summary statistics of overall scores, including means with associated 95% confidence intervals, are presented. The primary intention-to-treat (ITT) analysis, whereby participants are retained in the arms to which they are randomised, was based on multivariable linear regression adjusted for baseline continuous SNOT-22 score and the stratification variables sex and baseline severity categories based on the NOSE scale (model 1). Goodness of fit for model 1 was assessed by a series of plots of residuals. We considered a two sided P<0.05 to be statistically significant.

The planned population defining analyses included an ITT analysis, a per protocol analysis, and a per treatment analysis.

*ITT analysis*—this analysis was limited to those participants who completed the primary outcome SNOT-22 within the specified window of six months (−2 to 4 weeks). This analysis was termed the compliant ITT analysis.

*Per protocol analysis*—this analysis was performed as per the ITT analysis, restricted to those participants randomised to, and undergoing, septoplasty within the specified 12 weeks after randomisation and who completed the primary outcome within the specified window, compared with those randomised to receive medical management who remained within that group and did not cross over to surgery.

*Per treatment analysis*—this analysis was performed as per the ITT analysis, comparing participants who underwent septoplasty, regardless of initial randomisation group, at any time during follow-up (but >10 weeks before the primary outcome measure at six months, to ensure postoperative inflammation had settled), and all those treated with medical management, both regardless of initial randomisation group, with participants who completed the primary outcome within the specified window.

We explored the impact of the recruitment site on the primary outcome analysis by inserting the centre as a random effect with the mixed effect model.

Analyses of the secondary outcome measures followed the same primary multiple linear regression modelling. Although inferior turbinate reduction was not a randomised intervention, its effect on the primary outcome was explored as a planned covariate in the linear regression model.

Further planned multivariable linear regression sensitivity of the ITT analyses considered adjustment for other important baseline factors in the regression model. To utilise the full information from the continuous measure, model 2 adjusted for continuous baseline score on the NOSE scale rather than the three categories used at baseline. Model 3 included age, ethnicity, recruitment site (as a random effect), smoking history, double ordinal airway subjective scale, endoscopy findings (location of deviation, severity of airway blockage, and enlargement of the inferior turbinate), and four nasal patency variables. These variables were initially regressed univariately against the primary outcome measure to screen for independent relatedness. Any variable with P>0.1 was included for consideration in further modelling based on forward selection. Non-linear continuous covariates were considered for fractional polynomial transformations, if appropriate.

The importance of baseline severity within the ITT population, as a continuous distribution of score on the NOSE scale at randomisation, was further explored graphically using a subpopulation treatment effect pattern plot (STEPP) analysis.^14^ This analysis enabled the predicted point estimates of any treatment effect (with 95% confidence intervals) between the randomised groups to be displayed over the range of NOSE scale values (30-100), with the aim of further informing patient selection guidance and recommendations.

All data were analysed using STATA, version 16.

### Missing data

SNOT-22 questionnaires with up to 20% of items missing were imputed with the average of the completed questions used for missing items. A sensitivity analysis using multiple imputation was also carried out that included all participants who provided consent and were randomised, including those with missing SNOT-22 scores at the primary endpoint. Baseline variables found to be predictive of missing data status were included in multiple imputation equations. To make the missing at random assumption as plausible as possible, the multiple imputation equation included baseline data on sex, NOSE scale categories, baseline SNOT-22 score, and predictors of missing data. Overall, 1000 multiple imputation datasets were created in STATA16 using chained equations. A conservative approach was adopted, and treatment group was included in the imputation model.

### Sample size calculation

The sample of 378 participants was based on 90% power to detect a minimal clinically important difference of 9 points on SNOT-22,^8^ assuming a conservative standard deviation of 24 points^15^ and a type 1 error rate of 5%, and to allow for 20% drop-out.

### Patient and public involvement

Patient and public involvement was integrated throughout the design and conduct of the trial. More than 20 patients were consulted about the primary outcome measure, with most favouring the items covered by SNOT-22 over NOSE scale questionnaire. Patients’ views were integrated into the design to encourage any crossover to septoplasty to occur after six months. All patient facing documents were reviewed for acceptability.

## Results

Between 26 January 2018 and 5 December 2019, 378 eligible participants (67% men) were randomised. Baseline personal characteristics were balanced across treatment groups (table 1).

**Table 1:** Baseline characteristics of recruited participants. Values are number (percentage) unless stated otherwise

Characteristics	Septoplasty (n=188)	Medical management (n=190)	Total (n=378)
**Sex**			
Male	126 (67)	127 (67)	253 (67)
Female	62 (33)	63 (33)	125 (33)
**Age (years)**			
Median (IQR)	38 (27.5, 51)	37 (28, 50)	38 (28, 50)
Mean (SD)	40.3 (14. 9)	39.4 (13.9)	39.8 (14.4)
Range	18-79	18-80	18-80
**Ethnicity**			
White	169 (90)	165 (87)	334 (88)
Asian (Indian/Pakistani/Bangladeshi ancestry)	13 (7)	14 (7)	27 (7)
Other ethnic origin	4 (2)	11 (6)	15 (4)
Missing	2 (1)	0 (0)	2 (<1)
**Baseline NOSE score (continuous)**			
Median (IQR)	70 (60, 82.5)	70 (60, 85)	70 (60, 85)
Mean (SD)	69.9 (17.4)	71.3 (17.3)	70.6 (17.4)
Range	30-100	30-100	30-100
**Baseline NOSE categories**			
Moderate	30 (16)	32 (17)	62 (16)
Severe	89 (47)	89 (47)	178 (47)
Extreme	69 (37)	69 (36)	138 (37)

IQR=interquartile range; NOSE Nasal Obstruction and Symptom Evaluation scale; SD=standard deviation.

The onset of the covid-19 pandemic resulted in the suspension of all face-to-face clinics from 30 March 2020, therefore remaining trial participants were invited to complete the primary outcome six month SNOT-22 measure (n=8, 2%) and the secondary outcome 12 month SNOT-22 measure (n=25, 7%) remotely. By then recruitment had been achieved and all trial interventions had been completed. Information on adverse events was collected remotely by telephone at the six month and 12 month follow-ups. Only 66 participants in the septoplasty group and 72 in the medical management group completed the 12 month objective flow measures.

Four SNOT-22 questionnaires had missing items at baseline—two in each treatment group (1%), five at the primary six month endpoint (2%) and four at the final 12 month data collection point (1%). All of these were imputed as each had <20% missing. One participant had one item missing from the NOSE scale questionnaire (20%) at the six month follow-up. This was also imputed, using the mean of the other four responses.

### Treatment received

**Fig 1:**
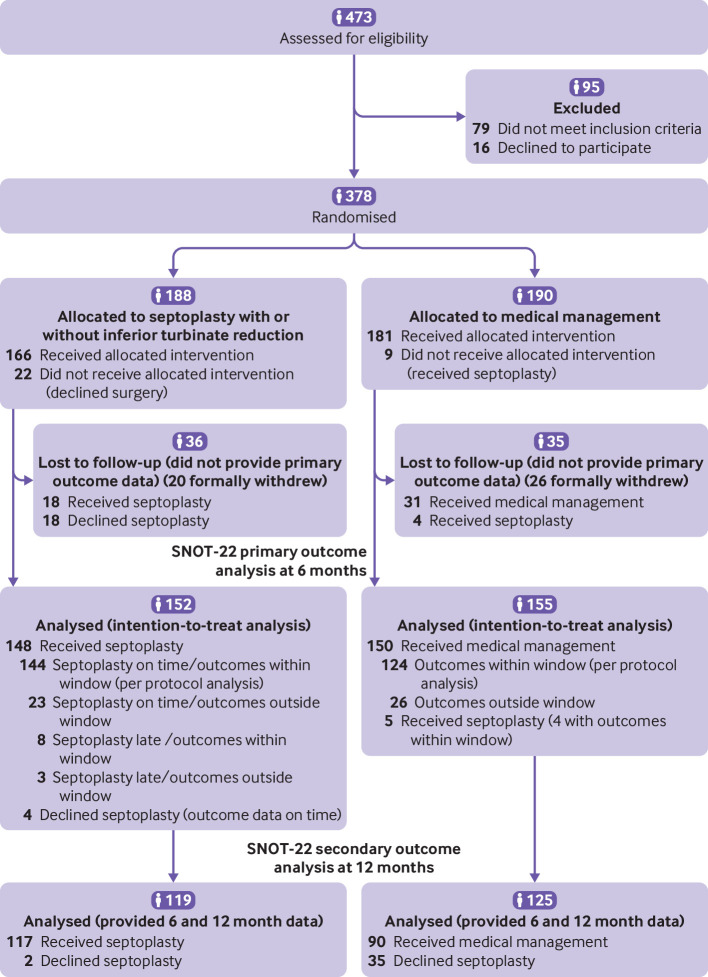
Flow of participants through study

Of the 378 participants randomised, 188 were assigned to receive septoplasty and 190 were assigned to receive medical management (fig 1). Overall, 307 participants (81%) were included in the ITT analysis of the primary outcome. In the ITT septoplasty group, four (3%) participants did not receive the surgical intervention; in the ITT medical management group, five (3%) participants received septoplasty. Consultant surgeons carried out 128 of 166 (77%) of the septoplasties for participants randomised to the septoplasty arm, who received the intervention. Associate specialists carried out 17 (10%) of the septoplasties, and other grades carried out 16 (10%). The records of five (3%) failed to record the grade of the most senior operative surgeon. In total, 112 out of 148 participants (76%) randomised to septoplasty and who received the intervention in the ITT analysis, underwent surgery within eight weeks after randomisation (see supplementary analysis figure S1). Twenty five (17%) underwent surgery within eight and 12 weeks after randomisation. One hundred and twenty two (64%) of the 190 participants randomised to medical management completed the drug adherence feedback, of whom 87 (71%) used nasal steroid spray >90% of the time, 101 (83%) used it >75%, and 17 (14%) had rarely used the drug.

### Primary outcome analysis

Patient reported symptoms improved in both groups. In those participants randomised to receive septoplasty in the ITT population (n=152), mean SNOT-22 scores were 44.5 (95% confidence interval 41.1 to 47.8) at baseline and 19.9 (17.0 to 22.7) at six months. The corresponding scores for those participants randomised to receive medical management in the ITT population (n=155) were 44.1 (40.8 to 47.4) and 39.5 (36.1 to 42.9). Baseline SNOT-22 scores were approximately symmetrically distributed about the mean and were not transformed for the analysis (see supplementary material figures S2 and S3). In the primary ITT analysis, participants randomised to the septoplasty group reported on average 20.0 units greater improvement in symptoms compared with those randomised to the medical management group, as measured by SNOT-22 (95% confidence interval improvement 16.4 to 23.6, P<0.001; table 2). The lower limit of the 95% confidence interval is greater than the minimal clinically important difference of 9 points. With the presence of baseline SNOT-22 in the model (table 2), the stratification variables did not appear to have a major influence on the primary outcome. The primary outcome results were unchanged when further baseline variables were considered in the analysis, including the recruitment site when entered into model 2 as a random effect (see supplementary material tables S1 and S2 and figure S4).

**Table 2:** Patient reported SNOT-22 score at six months (primary outcome) adjusted for baseline SNOT-22 score and stratification variables sex and NOSE categories in 307 participants in intention-to-treat population

	Coefficients (95% CI)	P value
Septoplasty arm (reference category medical management)	−20.01 (−23.63 to −16.40)	<0.001
Baseline SNOT-22 score	0.50 (0.39 to 0.60)	<0.001
Male sex (reference category female sex)	−0.55 (−4.38 to 3.27)	0.78
NOSE severity (reference category moderate):		
Severe	1.98 (−3.85 to 7.81)	0.50
Extreme	5.81 (−1.00 to 12.62)	0.094
Constant	14.95 (8.48 to 21.43)	<0.001

NOSE=Nasal Obstruction and Symptom Evaluation scale; SNOT-22=Sino-Nasal Outcome Test-22.

Adjusted R^2^=0.472. Probability *F*_5,301_=55.69, P<0.001.

**Fig 2:**
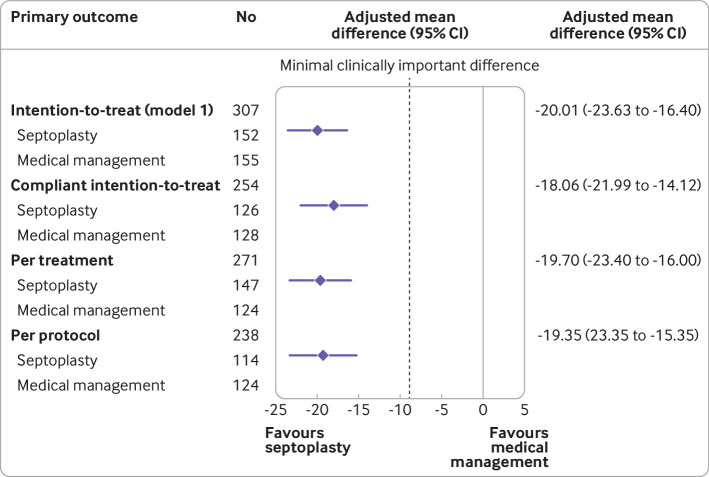
Forest plot of primary analysis and sensitivity analysis for intention-to-treat population. CI=confidence interval

The planned population defining analyses all showed similar results and supported the primary ITT analysis (fig 2). After multiple imputation to account for missing data (n=378), participants randomised to the septoplasty group (n=188) reported on average a greater improvement of 20.0 units over those randomised to the medical management group (n=190) (95% confidence interval improvement 16.4 to 23.7, P<0.001) supporting the primary ITT analysis

### Secondary outcomes

**Fig 3:**
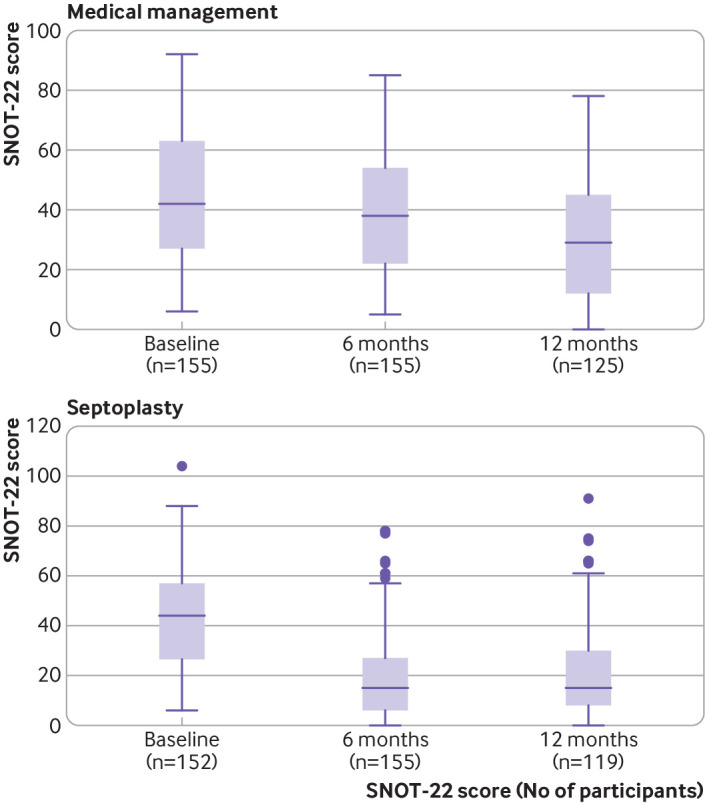
Box plots showing summary statistics for SNOT-22 scores in intention-to-treat groups. Box represents middle 50% of data (lower quartile to upper quartile), horizontal line in box shows median (50th centile), whiskers show data that fall within 1.5× interquartile range, and points show data that fall outside these limits. SNOT-22=Sino-Nasal Outcome Test-22

The improvement in SNOT-22 scores in the septoplasty group remained at 12 months but was less noticeable compared with those in the medical management group (fig 3). The mean SNOT-22 score in the septoplasty group was 21.2 (95% confidence interval 17.7 to 24.6) at 12 months, whereas the corresponding values in the medical management group were 30.4 (26.6 to 34.3). Participants randomised to the septoplasty group (n=119) reported on average 10.1 units greater improvement in symptoms at 12 months compared with those randomised to the medical management group (n=125), as measured by SNOT-22 (95% confidence interval improvement 5.6 to 14.5, P<0.001).

Supplementary table S3 shows that the secondary outcomes measured at six months improved more in the septoplasty group than in the medical management group, including the objective flow measures. A noticeable improvement in SNOT-22 scores was observed between six and 12 months for 29 of the 37 participants randomised to receive medical management and who chose to undergo septoplasty after six months, restricted to those who provided 12 months’ data. The SNOT-22 scores for these 37 participants had not improved between baseline and six months (see supplementary figure S5). The analysis between the groups in the 12 month objective assessments was affected by lack of face-to-face assessments during the covid-19 pandemic.

For the ITT population (n=307) mean scores on the SF-36 physical component summary and mental component summary at six months were 2.74 (95% confidence interval 1.23 to 4.25) and 4.39 (2.43 to 6.36) units higher, respectively (better health), in the septoplasty group than in the medical management group (adjusted for baseline severity and stratification variables, P<0.001; see supplementary tables S5-S8 for full details).

**Fig 4:**
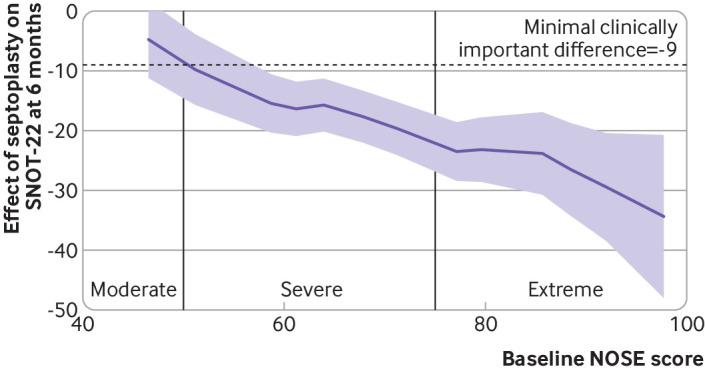
Outcome data using subpopulation treatment effect pattern plot to assess individual changes in SNOT-22 scores from baseline to six months. Purple line shows average effect of being randomised to septoplasty for those with specific NOSE scores at baseline and shading represents 95% confidence intervals. Minimal clinically important difference is 9 points on SNOT-22. NOSE=Nasal Obstruction and Symptom Evaluation; SNOT-22=Sino-Nasal Outcome Test-22

The STEPP analysis shows that for the 18 participants with moderate NOSE scores at baseline the average improvement in SNOT-22 score was around 5 units compared with medical management (fig 4). The 77 participants with scores around 60 had an average improvement of around 15 units compared with medical management, whereas the 57 participants with extreme scores at baseline improved by as much as 30 units compared with medical management. No major differences were noted in the STEPP analysis graphs between male and female participants. When included in the primary outcome multiple regression model, baseline NOSE scores scored on a continuous scale were positively related to the six month SNOT-22 scores (P=0.041, see supplementary table S1).

For the 148 participants who underwent septoplasty and had SNOT-22 scores at both baseline and six months, there was no difference in primary outcome scores between those chosen to receive inferior turbinate reduction and those chosen not to receive inferior turbinate reduction: 2.8 points difference (95% confidence interval −2.78 to 8.35 points difference; P=0.33).

No other clinically relevant factors showed a statistically significant association with the primary outcome of patient reported SNOT-22 score at six months (see supplementary tables S1 and S2). The primary outcome SNOT-22 scores were not altered appreciably by the reported adherence rates in participants treated with medical management (see supplementary figure S6).

### Safety

Table 3 summarises the reported serious adverse events and adverse events. Specific complications related to septoplasty (including participants from both randomised groups who underwent septoplasty and including both serious adverse events and adverse events in table 3) were seven readmissions with bleeding (4% of 174 participants who underwent septoplasty), 20 infections requiring antibiotics (12%), 19 reported events of altered sense of smell (11%), 18 events of upper teeth numbness (11%), 17 reported changes in the appearance of the nose (10%), 7 clinician observed adhesions (4%), and 6 clinician observed septal perforations (3%). No participants required repeat surgery.

**Table 3:** Reported serious adverse events and adverse events among all participants

Adverse events	Septoplasty group (n=188)	Medical management group (n=190)	Total
Total reported serious adverse events	14	9	23
Anaesthetic complication	3	2	5
Infection	2	2	4
Postoperative bleeding	5	1	6
Vasovagal episode	2	0	2
Multi-pill overdose	0	3	3
Trauma unrelated to the trial	0	1	1
Overnight hospital admission after surgery	2	0	2
Total No of reported adverse events*	132	95	227
Deaths	0	0	0

## Discussion

Septoplasty results in significantly greater improvement in patient reported SNOT-22 scores at six months compared with a regimen of nasal steroid and saline sprays, and this improvement is sustained to 12 months. We therefore recommend that adults presenting with nasal obstruction associated with a deviated nasal septum, in the absence of coexistent nasal or sinus disease and with a baseline NOSE score >30, can reliably be offered surgery. The per protocol and per treatment analyses corroborated the ITT results, confirming the greater improvement in patient reported outcomes of those participants who received surgery compared with those treated medically.

The STEPP analysis provided evidence of the increasing improvement in SNOT-22 scores as baseline NOSE score severity increased. This effect was observed in both male and female participants. This analysis can enable clinicians to quantify expected improvements in outcomes of patients considering septoplasty, predicated on baseline NOSE score. The STEPP analysis, in parallel with an understanding of the potential risks associated with both medical treatment and surgery, substantially improved the quality of information available to the clinician and patient in the decision making process around septoplasty.

In 2014 when the National Institute for Health and Care Research commissioned this trial, no high level evidence existed for surgery compared with medical management for nasal obstruction associated with septal deviation. No specific guidance was available as to who should be referred for consideration of surgery, or the likely outcomes. The intervention was practice based, not evidence based. To our knowledge, in 2019 van Egmond et al reported the first randomised clinical trial assessing septoplasty.^16^ Guidance on referrals has not been updated since its publication.^4^ The study by van Egmond et al reached similar conclusions to that of the current study in a trial of 203 participants in the Netherlands randomised to receive either septoplasty or non-surgical management. Some important distinctions need to be mentioned between these two trials conducted in different healthcare systems: in the study by van Egmond et al, non-surgical management was not defined, whereas in the current study we specified a standardised medical management arm, which we considered offered the optimal medical treatment for any intranasal mucosal inflammation. The primary endpoint in the study by van Egmond et al was 12 months, and by that stage 30% of the non-surgical arm had crossed over to the surgical arm. The shorter time point of six months for our primary outcome aimed to minimise crossovers from the medical management arm while allowing sufficient healing time after surgery to give a true representation of any clinical difference between arms in an ITT analysis. In addition, we used a disease specific patient reported outcome measure rather than a general quality of life measure for the primary outcome.

We estimated a superiority for septoplasty of 20 points on SNOT-22 at six months compared with 9.7 points at 12 months in the study by van Egmond et al. Their data transformation and variable non-surgical arm treatments make direct comparison with the current study difficult. The two clinical trials report concordant results in showing that septoplasty is clinically effective. The current study substantially enhances understanding of the role of quantitative patient reported data in the selection of patients for surgery by showing that the degree of improvement in symptoms (as measured by SNOT-22) is closely related to baseline severity stratification (as measured by NOSE score). This improvement in SNOT-22 score remained across each of the four SNOT-22 subdomains— nose, sleep, ear/facial pain, psychological. The impact of septal surgery in improving SNOT-22 scores is also found in studies of snoring^17^ and eustachian tube dysfunction.^18^

In the current study, although a greater and sustained improvement was observed in the surgical arm, we did note a potentially clinically important improvement in SNOT-22 scores in the medical management arm of 9.1 points between six and 12 months. In the ITT analysis, some of the improvement at 12 months in the medical management group may have been affected by 37 participants who were randomised to receive medical management undergoing septoplasty after six months. The primary outcome, SNOT-22 score at six months, did not improve from baseline in these 37 participants, suggesting that these participants undergoing septoplasty had an important impact on the medical management group.

The benefits of septoplasty were also seen in the secondary outcome measures, including clinical airflow assessment, patient reported outcome measures, and objective measurements of peak nasal inspiratory flow and rhinospirometry. Absolute subjective double ordinal airway subjective scale, the subjective comparator of the worse versus the better nostril airflow, revealed a substantial treatment related shift from predominantly unilateral nasal airflow to equal airflow through both nostrils, more noticeable in magnitude in the surgical arm than in the medical management arm at both six and 12 months. It may be a useful tool to audit surgical outcomes, or future trials of nasal airway surgery.

The results of the current study infer that turbinate surgery added no additional improvement to septoplasty alone. The decision to perform turbinate surgery was, however, pragmatic and left to the discretion of the operating surgeon. The results for inferior turbinate reduction may not be generalisable to other techniques or to bilateral reduction practices. The study by van Egmond et al similarly reported no additional benefit of turbinate surgery.^16^ As in the current study, turbinate reduction was a surgeon led decision and not a randomised intervention. Neither clinical trial can draw firm conclusions on the impact of inferior turbinate reduction in combination with septoplasty. A recent single centre trial randomised participants undergoing septoplasty to inferior turbinate reduction or no inferior turbinate reduction.^19^ This study reported a statistically significant and sustained additional benefit of inferior turbinate reduction, as measured using the NOSE score, implying that further multicentre clinical trials should be considered to define the impact of inferior turbinate reduction in combination with septoplasty.

### Limitations of this study

The study has several limitations. At baseline, more than 80% of patients had NOSE scores in the severe or extreme category. This may reflect the population currently referred to secondary care. However, the conclusions for these two subgroups were strong. The benefits for those participants with moderate baseline symptoms was less clear, whereas those with moderate NOSE scores at baseline did not report an improvement that reached the minimal clinically important difference of 9 points on SNOT-22—only 18 participants were included in this subgroup of the STEPP analysis.

Nasal obstruction is a non-specific symptom with many underlying possible causes (chronic rhinosinusitis, allergic rhinitis, non-allergic rhinitis, nasal valve dysfunction). As a pragmatic trial, the current study did not seek to diagnose or treat these conditions before randomisation. Also, surgical interventions were performed by experienced surgeons. In NHS practice, septoplasty is often performed by junior trainee surgeons, albeit many are supervised by more senior colleagues. The evidence is limited for septoplasty outcomes and surgeon experience. However, previous studies have found no association between grade of surgeon and septoplasty outcomes when assessing the need for revision surgery^20^ or the postoperative appearance of the septum.^21^ Covid-19 had a considerable impact on the current trial. All forms of airway clinical assessment and objective measurements of nasal patency were suspended from March 2020 onwards. As a result of the smaller numbers of participants, this may have had an impact on the precision of the statistical results. All other patient reported outcome measures were collected remotely during the pandemic, ensuring that we could report the trial results reliably.

The current trial did not assess, and therefore cannot define, what should constitute an appropriate trial of medical management for patients and clinicians to consider before septal surgery is discussed. Participants in the medical management group reported an improvement in symptoms with mometasone nasal steroid and saline sprays. Participants with an element of underlying rhinitis contributing to the nasal obstruction in addition to the septal deviation may have responded to this treatment. Consensus opinion from the US recommends a course of medical management for four weeks before septoplasty is considered.^22^

### Conclusions

Septoplasty is a superior treatment for nasal obstruction associated with septal deviation compared with a defined regimen of nasal steroid and saline sprays. Baseline NOSE scores can estimate the likely improvement in symptoms and guide decision making for patients and clinicians. The authors recommend that adults presenting with nasal obstruction associated with a deviated nasal septum should be offered septoplasty.

**What is already known on this topic**

In 2014, the UK National Institute for Health and Care Research commissioned a randomised controlled trial to address the lack of high quality evidence to support nasal septoplasty
A study in 2019 reported higher levels of general quality of life at 12 months in participants after septoplasty compared with those who received variable non-surgical management
In that study, score on the Sino-Nasal Outcome Test (SNOT-22) was a secondary outcome, with greater improvement observed in participants after septoplasty

**What this study adds**

Septoplasty is a clinically effective treatment for nasal obstruction associated with septal deviation compared with a defined regimen of nasal steroid and saline sprays
Participants who underwent septoplasty had greater improvement in nasal obstruction and quality of life, as measured using the SNOT-22 questionnaire
However, people with a deviated nasal septum and at least moderate nasal obstruction do tend to improve over time with medical treatment

## Data Availability

The chief investigator and collaborators will act as custodians of the trial data and manage data sharing requests through ReShare, a UK online data repository recommended by the funder. Parties interested in data sharing will directly contact the chief investigator and collaborators and complete a project proforma and provide the rationale and the information required and comply with the regulation of responsibilities of users. If agreed to be appropriate, requested anonymised data will be made available. An access advisor who is independent of the trial team will periodically review access decisions.

## Web extra

Extra material supplied by authors

Supplementary information: Inclusion and exclusion criteria, figures S1-S6, and tables S1-S8

## Notes

Contributors: All authors have contributed to writing, reviewing, and revising the manuscript. SC and JOH are joint first authors. SC is the corresponding author and NAIROS chief investigator.TF, DS, and DT performed the trial statistical analysis. TH and LT performed the trial health economic evaluation. NR and KL performed the qualitative research. KR, EC, and AS were the trial management team. NW managed the trial database. LR, JD, and CW performed the qualitative research that guided trial recruitment. AB and MD devised the nasal patency methodology and performed the related data analysis. DH and JW were co-applicants and contributed to the trial design and participant recruitment. YO contributed to the trial methodology design. JM acted as a co-applicant and primary care lead. QG, NK, SK, SL, SM, and PN were clinical co-applicants and contributed to participant recruitment. SC and JOH, as guarantors, accept full responsibility for the work and/or the conduct of the study, had access to the data, and controlled the decision to publish. The corresponding authors attests that all listed authors meet authorship criteria and that no others meeting the criteria have been omitted.

The lead authors (SC and JOH) affirm that the manuscript is an honest, accurate, and transparent account of the study being reported; that no important aspects of the study have been omitted; and that any discrepancies from the study as planned have been explained.

Dissemination to participants and related patient and public communities: A lay summary of results will be available on the NAIROS website. Access to the Health Technology Assessment report will also be available through the trial website. Members of the patient and public involvement focus groups will review results and they will be involved in writing lay summaries of results for dissemination to relevant patient groups.

Provenance and peer review: Not commissioned; externally peer reviewed.
