# Supplementary material for: Clinical effectiveness of septoplasty versus medical management for nasal airways obstruction: multicentre, open label, randomised controlled trial
Source: BMJ. 2023 Oct 18;383:e075445. doi: 10.1136/bmj-2023-075445 (PMC10583133; doi:10.1136/bmj-2023-075445)
Supplement: Supplementary file 1 — Supplementary information: Inclusion and exclusion criteria, figures S1-S6, and tables S1-S8 [file cars075445.ww.pdf]

Supplementary Material

Inclusion Criteria

- Adults aged or over 18 years.
- Baseline Nasal Obstruction and Septoplasty Effectiveness (NOSE) ≥ 30.
- Septal deflection visible via nasendoscopy.
- Capacity to provide informed consent / complete trial questionnaires.

Exclusion Criteria

- Prior septal surgery.
- No cosmetic / revision procedures
- No open septoplasty approach allowed
- Systemic inflammatory disease / use of oral steroid treatment within the previous two weeks.
- Granulomatosis with polyangiitis.
- Naso-endoscopic evidence of unrelated associated pathology
- Intranasal recreational drug use within the past six months.
- Breast feeding, pregnancy or intended pregnancy for duration of involvement in the trial.
- Bleeding diathesis.
- Therapeutic anticoagulation.
- Contraindication to general anaesthesia.
- Immuno-compromise.
- External bony deformity.

Figure S1 displays time to surgery for participants randomised to the septoplasty arm and for whom primary endpoint data was collected (ITT population).

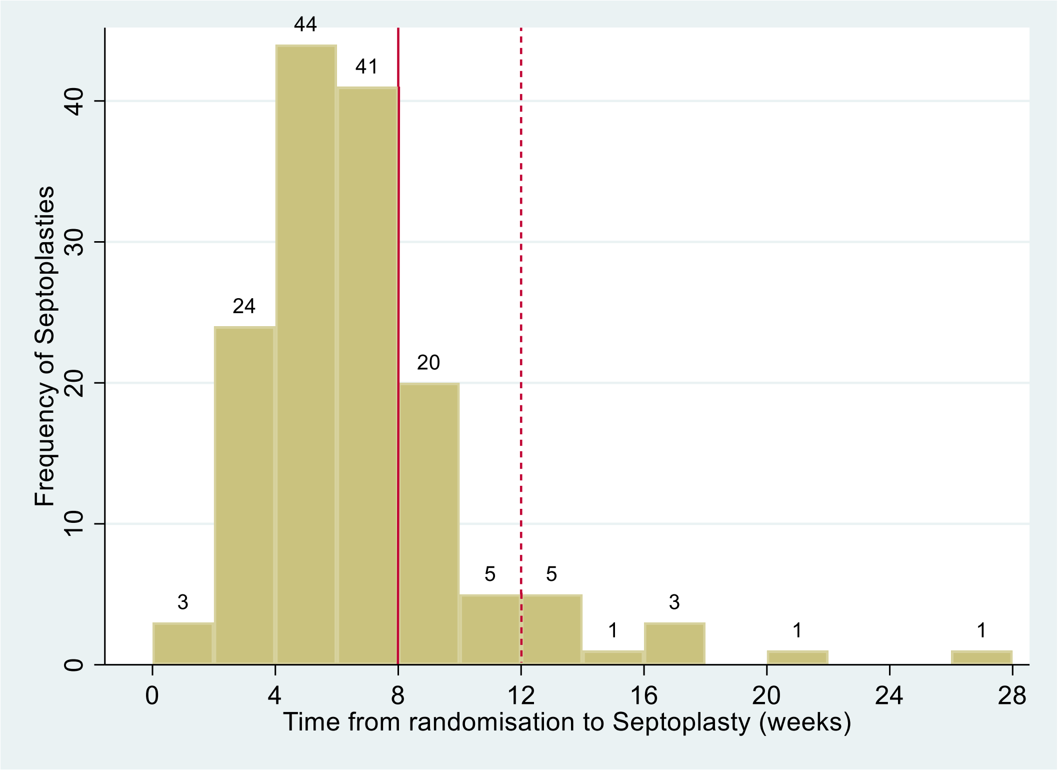

The red solid line shows the eight- week compliance window for septoplasty. The red hatched line shows the additional four- week window which allowed for delays in delivering septoplasty in extenuating circumstances.

Figure S2 Baseline SNOT22 scores for the ITT population – histogram and overlying normal curve

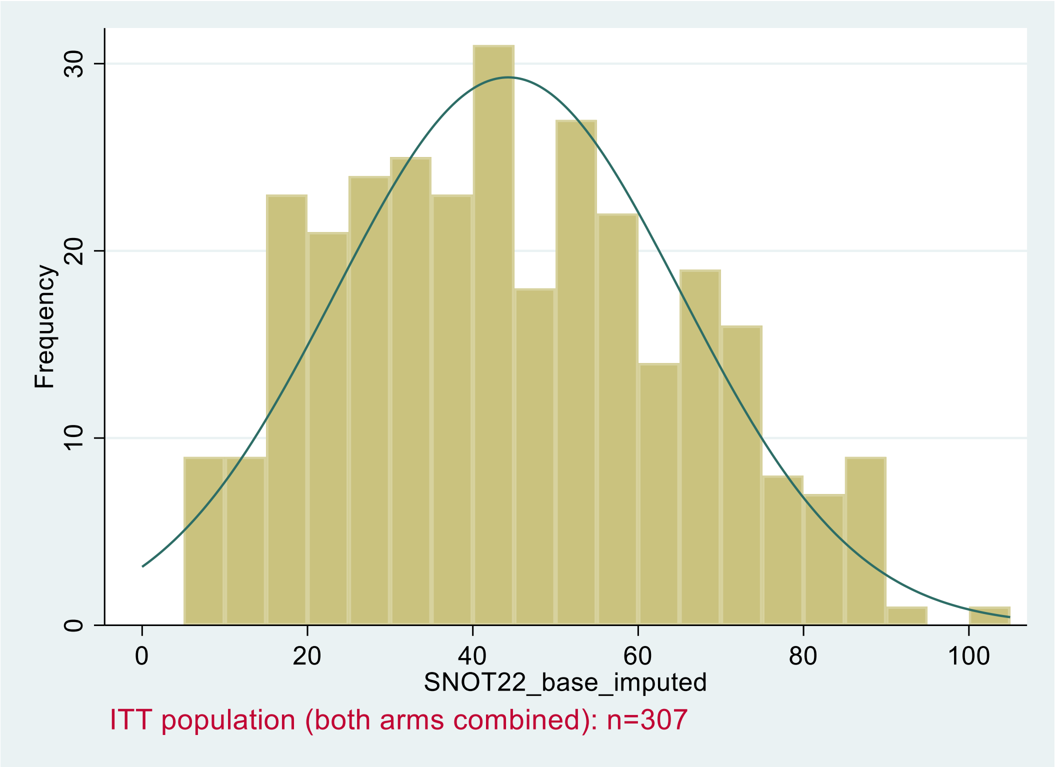

Figure S3 Series of plots of residuals

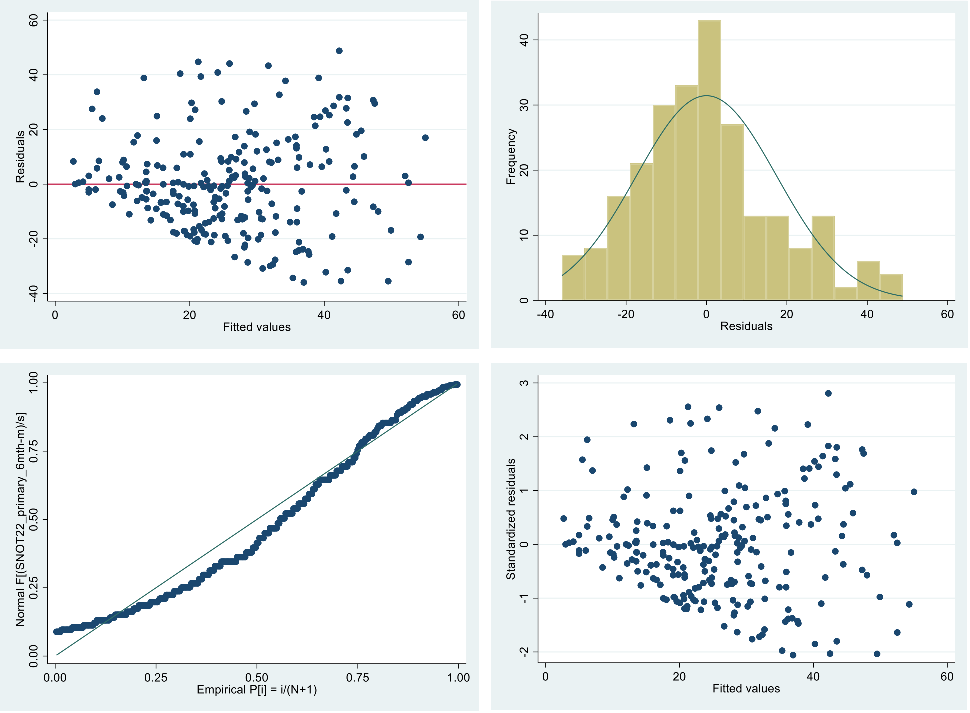

Goodness of fit for model 1 was assessed by a series of plots of residuals. The residuals appeared normally distributed with no apparent pattern in fitted values versus residuals, fewer of the standardised residuals fell outside the range (-2, +2).

Residual analysis showed that the residuals were approximately normally distributed, hence validating our regression model and the results it produced.

Table S1 Model 2 - SNOT22 at 6 months primary endpoint adjusted for baseline SNOT22, stratification factor gender and continuous baseline NOSE score - ITT population

Primary outcome measure SNOT22 at 6 months	coeff	P value	95% Confidence interval coefficient	
			Lower	upper
Arm: (ref category: medical management)<br>Septoplasty	-19.694	<0.0001	-23.298	-16.090
Baseline SNOT22	0.477	<0.0001	0.364	0.590
Gender (ref category: female)<br>male	-0.751	0.699	-4.569	3.067
NOSE severity (continuous)	0.146	**0.041**	0.006	0.286
Constant	8.501	0.054	-0.147	17.149

N=307, adjusted R square=0.4743. *Prob>=F=<0.0001*

Table S2 Model 3 - SNOT22 at 6 months primary endpoint adjusted for baseline SNOT22, stratification factor gender and continuous baseline NOSE score - after forward selection applied to include reciprocal tidal volume (Arm forced into model)

Primary outcome measure SNOT22 at 6 months	coeff	P value	95% Confidence interval coefficient	
			Lower	upper
Arm: (ref category: medical management)<br>Septoplasty	-20.426	**<0.001**	-24.203	-16.650
Baseline SNOT22	0.454	**<0.001**	0.329	0.578
Gender (ref category: female)<br>male	1.158	0.577	-2.929	5.245
NOSE severity (continuous)	0.134	**0.076**	-0.014	0.283

Reciprocal of median tidal volume	1446.909	**0.003**	510.134	2383.685
Constant	6.198	0.182	-2.919	15.315

N=264, adjusted R square=0.4859. *Prob>=F=<0.0001*

Figure S4 Forest Plot to show the effects of baseline variables within the primary outcome analysis

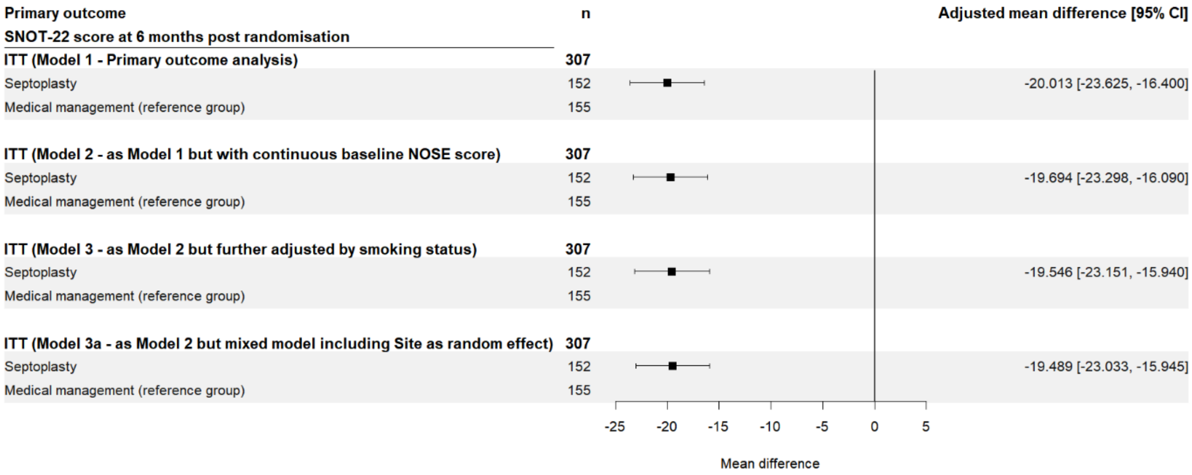

Table S3 Primary and Secondary Outcomes Descriptive Statistics for the ITT analysis

		**Septoplasty +/- IT reduction**			**Medical Management**			**Difference between group means at 6 months (p =)***	**Difference between group means at 12 months (p=)***
		**Baseline**	**6 months**	**12 months**	**Baseline**	**6 months**	**12 months**		
**SNOT 22**	n=	152	152	119	155	155	125		
	Mean	44.5	19.9	21.2	44.1	39.5	30.4	20.0	10.1
	95% CIs	41.1, 47.8	17.0, 22.7	17.7, 24.6	40.8, 47.4	36.1, 42.9	26.6, 34.3	16.4, 23.6 (p<0.0001)	5.6, 14.5 (p<0.0001)
**Nasal subscale**									
	Mean	17.0	7.8	8.2	16.3	14.5	11.3	#	#

	95% CIs	15.8, 18.2	6.7, 8.9	6.9, 9.5	15.1, 17.5	13.4, 15.6	9.9, 12.7	#	#
**Sleep subscale**									
	Mean	19.8	8.8	9.5	20.5	18.8	14.0	#	#
	95% CIs	18.0, 21.6	7.2, 10.4	7.7, 11.3	18.7, 22.2	16.9, 20.6	12.0, 16.0	#	#
**Otological subscale**									
	Mean	5.1	2.3	2.3	4.5	3.8	3.3	#	#
	95% CIs	4.3, 5.8	1.8, 2.8	1.6, 2.9	3.8, 5.2	3.2, 4.5	2.6, 4.0	#	#
**Emotional subscale**									
	Mean	2.7	1.0	1.2	2.8	2.4	1.8	#	#
	95% CIs	2.2, 3.1	0.7, 1.3	0.8, 1.6	2.3, 3.3	2.0, 2.8	1.4, 2.2	#	#
**NOSE**	n=	152	145	105	155	144	118		
	Mean	70.8	29.0	30.7	71.7	62.2	47.3	34.0	16.9
	95% CIs	68.1, 73.4	24.9, 33.1	25.7, 35.7	69.0, 74.4	58.3, 66.2	41.8, 52.7	28.6, 39.4 (p<0.0001)	9.6, 24.2 (p<0.0001)
**Absolute subjective DOASS**	n=	151	129	69	153	125	80		
	Mean	0.4	0.2	0.2	0.4	0.4	0.3	0.20	0.10
	95% CIs	0.3,0.4	0.1, 0.2	0.1, 0.2	0.3, 0.4	0.3, 0.4	0.2, 0.3	0.15, 0.25 (p<0.0001)	0.03, 0.17 (p = 0.006)

**PNIF**	n=	152	127	66	155	123	72		
	Mean	102	125.1	121.2	102.0	107.6	116.0	16.5	13.1
	95% CIs	93.8, 110.2	114.3, 136.0	106.0, 136.5	94.1, 109.8	99.0, 116.3	104.1, 127.9	6.4, 26.5 (p=0.001)	-0.23, 26.4 (p=0.054)
**Absolute Inhalational NPR**	n=	152	126	66	155	123	72		
	Mean	0.4	0.3	0.2	0.4	0.4	0.3	0.15	0.10
	95% CIs	0.4, 0.5	0.2, 0.3	02, 0.3	0.4, 0.5	0.4, 0.5	0.3, 0.4	0.08, 0.21 (p<0.0001)	0.019, 0.19 (p=0.016)

*results from analyses adjusting for baseline variables – these figures may not equate to the raw data presented in the other columns of this table.

Descriptive data only

Figure S5 Boxplots of SNOT-22 scores in the medical management arm differentiated by whether participants crossed over to receive septoplasty after 6 months (ITT population)

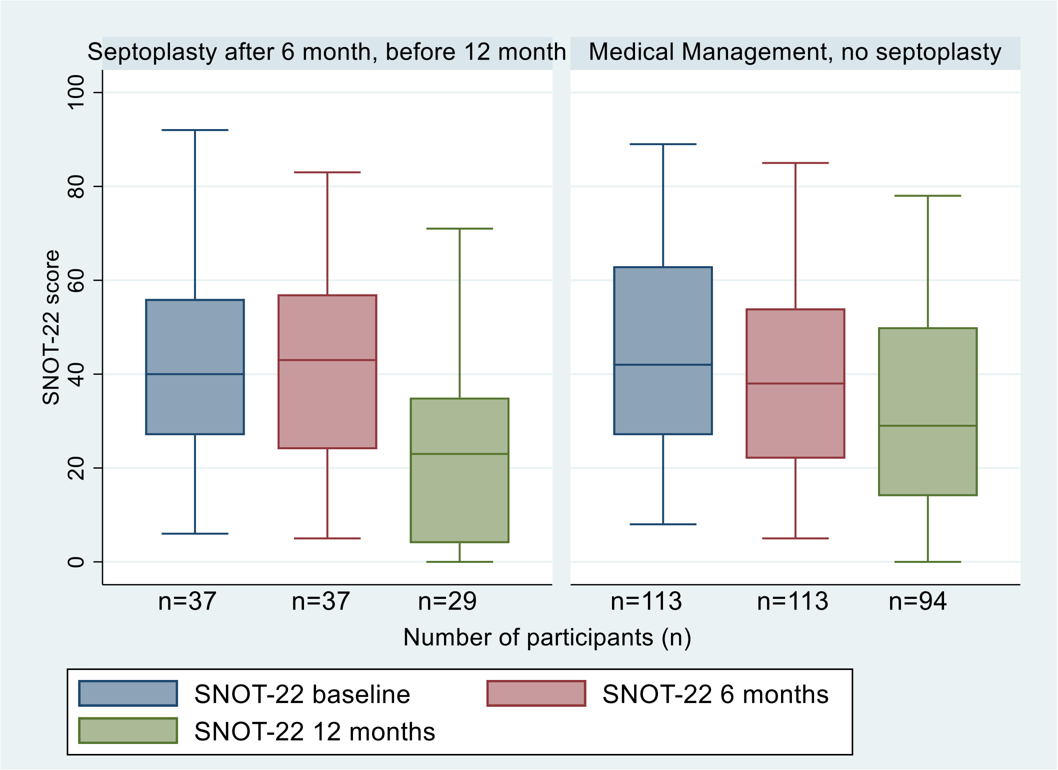

Table S4 Univariate analysis

Covariate	Number	Coefficient	SE	Test statistic	P value
Age (continuous)	307	0.085	0.087	0.98	0.328
Age log transform	307	2.674	3.356	0.80	0.426
Age complex transform (Age^3)	307	0.000	0.000	1.27	0.204
Ethnicity (ref: white)	272				
Asian	20	0.739	5.154	0.14	0.886
Other Asian	3	-5.477	12.915	-0.42	0.672
Other ethnic origin	10	1.695	7.163	0.24	0.813
Missing	2	N/A	N/A	N/A	N/A
Site (ref: site1 Newcastle)	62				
2- Great Yarmouth	19				**0.093**
3- Bradford	12				0.164
4- Wigan	12				0.595
5-Darlington	7				0.459
6-Plymouth	12				0.948
7-Dundee	17				**0.018**
8-Aberdeen	34				0.294
9-Stockportpt	13				0.272
10-London	12				0.805
11-Newport	11				**0.070**
12-Birmingham	12				0.577
13-Carlise	10				0.494
14-Aintree	17				**0.072**
15-Leeds	25				0.573

16-Lanarkshire	17				0.585
17-Salisbury	15				0.452
Smoking history (ref: smoker)	46				
Ex-smoker	88	-4.728	3.953	-1.20	0.233
Never smoked	173	-11.434	3.605	-3.17	**0.002**
Block (ref: bilateral)	128				
unilateral	179	-1.506	2.561	-0.59	0.557
Nasal trauma (ref: yes)	140				
No	167	-2.316	2.533	-0.91	0.361
Reduce turbinate (ref: yes)	230				
No	66	-5.410	3.081	-1.76	**0.080**
Not applicable	11				
Airway Block observer rated scale (ref:<=50%)	85				
>50%	222	-1.855	2.822	-0.66	0.511
Baseline Absolute Subjective DOASS	304	2.022	2.901	0.70	0.486
complex transform (Baseline Absolute Subjective DOASS ^-0.5)	304	0.788	1.449	0.54	0.587
Log Baseline Absolute Subjective DOASS	286	-0.238	1.803	-0.13	0.895
Baseline WORST DOASS	304	-1.257	0.617	-2.04	**0.043**
complex transform (Baseline WORST DOASS ^-0.5)	304	15.108	6.296	2.40	**0.017**
Log Baseline WORST DOASS	304	-4.689	2.003	-2.34	**0.020**
Baseline PNIF (post decongestant)	307	-0.048	0.025	-1.94	**0.054**
Log Baseline PNIF (post decongestant)	305	-0.312	1.694	-0.18	0.854
Complex transform (best is linear ^1)	N/A	N/A	N/A	N/A	N/A
Baseline absolute NPR (post decongestant)	307	3.465	4.114	0.84	0.400

Log Baseline absolute NPR (post decongestant)	305	-0.122	1.079	-0.01	0.991
Complex transform (^-1)	307	0.034	0.027	1.22	0.224
Baseline absolute Tidal breathing ratio (post decongestant)	306	0.552	4.082	0.14	0.893
Log Baseline absolute Tidal breathing (post decongestant)	305	0.485	1.164	0.42	0.677
Complex transform (^0)	306	0.686	1.170	0.59	0.558
**Medical physics derived endoscopy variables**					
Absolute NPR	273	-0.889	4.182	-0.21	0.832
Log Absolute NPR	273	0.204	1.220	0.17	0.867
Complex transform (^3)	273	-4.391	4.316	-1.02	0.310
Absolute decongestant NPR	270	-4.021	5.684	-0.71	0.480
Log Absolute decongestant NPR	165	-0.535	1.143	-0.47	0.640
Complex transform (^-2)	270	8.34×10^{-6}	7.60×10^{-6}	1.10	0.274
Total maximum flow rate	273	-0.005	0.005	-1.06	0.292
Log Total maximum flow rate	273	-3.407	2.699	-1.26	0.208
Complex transform (^-2)	273	280129.6	118248.3	2.37	**0.019**
Median tidal volume	264	-0.003	0.003	-1.17	0.245
Log Median tidal volume	264	-5.123	1.979	-2.58	**0.011**
Complex transform (^-1)	264	1995.859	627.311	3.18	**0.002**

Table S5Physical component summary (PCS) from the SF-36

	**Baseline**		**Six-month primary outcome**		**12-month follow-up**	
	Septoplasty (N=188)	Medical management (N=190)	Septoplasty (N=188)	Medical management (N=190)	Septoplasty (N=188)	Medical management (N=190)
ITT n (n/N)	152 (81%)	155 (82%)	152 (81%)	155 (82%)	152 (81%)	155 (82%)
ITT with completed SF36	149 (98%)	152 (98%)	141 (93%)	141 (91%)	103 (68%)	118 (76%)
Mean (SD)	50.4 (8.9)	50.7 (8.0)	53.2 (9.5)	50.9 (8.5)	52.7 (9.1)	52.0 (8.8)
95% CI about mean	(49.0, 51.8)	(49.4, 52.0)	(51.6, 54.8)	(49.5, 52.3)	(50.9, 54.5)	(50.4, 53.6)

Table S6 Mental component summary (MCS) from the SF-36

	**Baseline**	**Six-month primary outcome**	**12-month follow-up**

MCS	Septoplasty (N=188)	Medical management (N=190)	Septoplasty (N=188)	Medical management (N=190)	Septoplasty (N=188)	Medical management (N=190)
ITT n (n/N)	152 (81%)	155 (82%)	152 (81%)	155 (82%)	152 (81%)	155 (82%)
ITT with completed SF36	150 (99%)	152 (98%)	141 (93%)	142 (92%)	104 (68%)	118 (76%)
Mean (SD)	46.8 (11.4)	46.4 (11.4)	50.7 (10.5)	46.4 (11.2)	50.2 (10.2)	47.5 (11.1)
95% CI about mean	(45.0, 48.6)	(44.6, 48.3)	(49.0, 52.5)	(44.6, 48.3)	(48.2, 52.2)	(45.5, 49.5)

Table S7 SF36-PCS at 6 months primary endpoint adjusted for baseline SF36-PCS and stratification - ITT population

Primary outcome measure SF36-PCS at 6 months	coeff	P value	95% Confidence interval coefficient	
			Lower	upper
Arm: (ref category: medical management)				
Septoplasty	2.740	<0.0001	1.232	4.248
Baseline SF36-PCS	0.761	<0.0001	0.669	0.853
Gender (ref category: female)				
male	0.567	0.483	-1.024	2.158
NOSE severity (ref category: moderate)				
severe	0.069	0.953	-2.240	2.379
extreme	0.297	0.813	-2.180	2.774
Constant	11.827	<0.0001	6.453	17.202

N=276, adjusted R square=0.5155. *Prob>=F=<0.0001*

Table S8 SF36-MCS at 6 months primary endpoint adjusted for baseline SF36-MCS and stratification - ITT population

Primary outcome measure SF36-MCS at 6 months	coeff	P value	95% Confidence interval coefficient	
			Lower	upper
Arm: (ref category: medical management)				
Septoplasty	4.392	<0.0001	2.425	6.359
Baseline SF36-MCS	0.607	<0.0001	0.517	0.697
Gender (ref category: female)				
male	0.340	0.747	-1.729	2.408

NOSE severity (ref category: moderate)				
severe	-1.284	0.407	-4.329	1.761
extreme	-1.537	0.354	-4.797	1.723
Constant	18.964	<0.0001	13.364	24.565

N=278, adjusted R square=0.4298. *Prob>=F=<0.0001*

Figure S6 Primary outcome SNOT-22 at baseline and six-months for participants in the ITT medical management group who reported their compliance for the medication – greater than or less than 90% of the prescribed regimen.

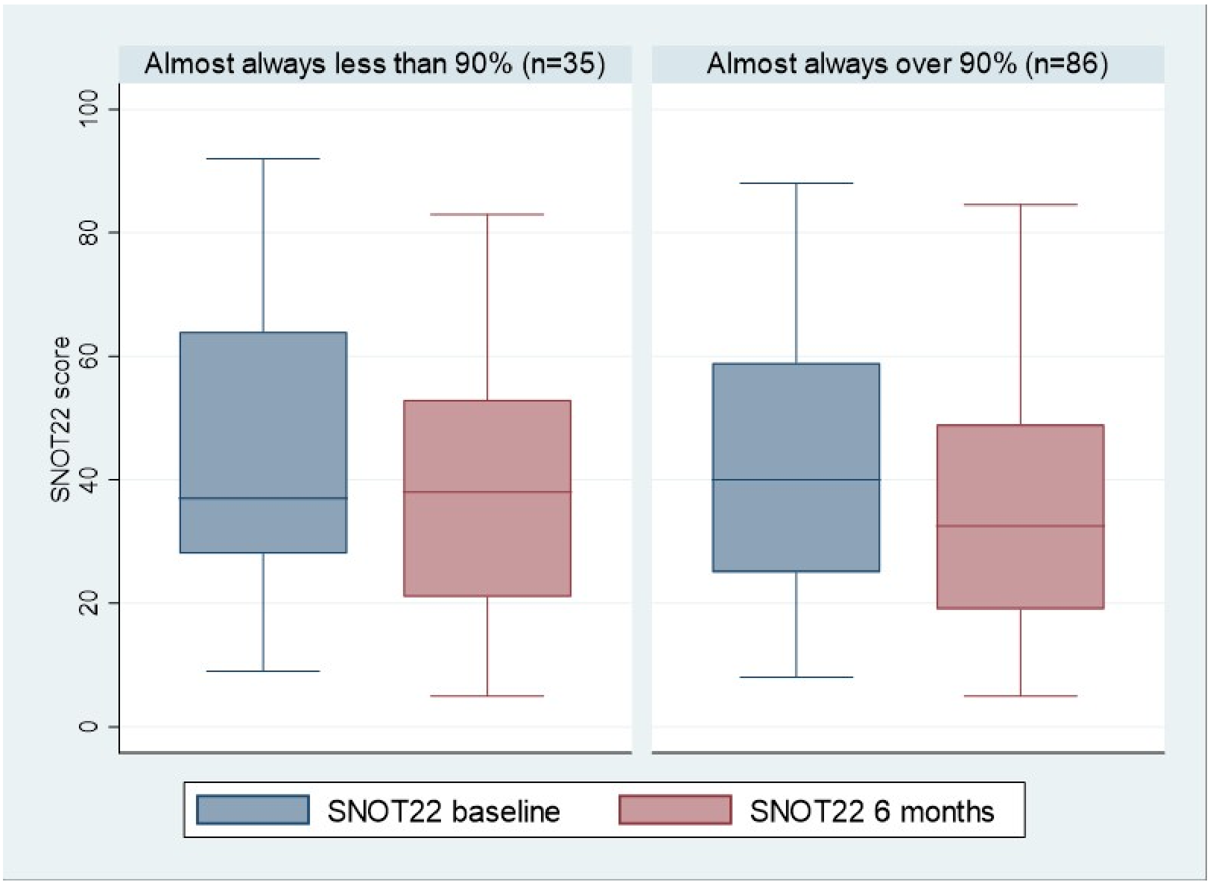

The above graph is based on n=121 in the ITT medical management group, who completed the compliance question. This is not n=122 as stated in the main manuscript as that included one participant who completed the medical compliance questionnaire but not the primary outcome SNOT-22 and was therefore not included in the ITT analysis.
